# Different faces of discrimination: perceived discrimination among homeless adults with mental illness in healthcare settings

**DOI:** 10.1186/1472-6963-14-376

**Published:** 2014-09-07

**Authors:** Anna Skosireva, Patricia O’Campo, Suzanne Zerger, Catharine Chambers, Susan Gapka, Vicky Stergiopoulos

**Affiliations:** Centre for Research on Inner City Health, Li Ka-Shing Knowledge Institute, Keenan Research, Centre, St. Michael’s Hospital, Toronto, Canada; Dalla Lana School of Public Health, University of Toronto, Toronto, Canada; Toronto People with Lived Experience Caucus, Toronto Local Advisory Committee, National Consumer Panel, Toronto, Canada; Department of Psychiatry, University of Toronto, Toronto, Canada

**Keywords:** Racial discrimination, Social discrimination, Homeless persons, Prejudice, Racism, Healthcare, Adult, Canada, Mentally Ill persons, Substance-related disorders

## Abstract

**Background:**

Research on discrimination in healthcare settings has primarily focused on health implications of race-based discrimination among ethno-racial minority groups. Little is known about discrimination experiences of other marginalized populations, particularly groups facing multiple disadvantages who may be subjected to other/multiple forms of discrimination. *Objectives*: (1) To examine the prevalence of perceived discrimination due to homelessness/poverty, mental illness/alcohol/drug related problems, and race/ethnicity/skin color while seeking healthcare in the past year among racially diverse homeless adults with mental illness; (2) To identify whether perceiving certain types of discrimination is associated with increased likelihood of perceiving other kinds of discrimination; and (3) To examine association of these perceived discrimination experiences with socio-demographic characteristics, self-reported measures of psychiatric symptomatology and substance use, and Emergency Department utilization.

**Methods:**

We used baseline data from the Toronto site of the At Home/Chez Soi randomized controlled trial of Housing First for homeless adults with mental illness (n = 550). Bivariate statistics and multivariable logistic regression models were used for the analysis.

**Results:**

Perceived discrimination related to homelessness/poverty (30.4%) and mental illness/alcohol/substance use (32.5%) is prevalent among ethnically diverse homeless adults with mental illness in healthcare settings. Only 15% of the total participants reported discrimination due to race/ethnicity/skin color. After controlling for relevant confounders and presence of psychosis, all types of discrimination in healthcare settings were associated with more frequent ED use, a greater - 3 - severity of lifetime substance abuse, and mental health problems. Perceiving discrimination of one type was associated with increased likelihood of perceiving other kinds of discrimination.

**Conclusions:**

Understanding the experience of discrimination in healthcare settings and associated healthcare utilization is the first step towards designing policies and interventions to address health disparities among vulnerable populations. This study contributes to the knowledge base in this important area.

**Trial registration number:**

This study has been registered with the International Standard Randomized Control Trial Number Register and assigned ISRCTN42520374.

## Background

Discrimination can be defined as the prejudicial and/or distinguishing treatment of an individual based on their actual or perceived membership or certain characteristics, such as race, ethnic origin, gender, sexual orientation, age, immigration status, income, medical condition or mental or physical disability. It can be conveyed through opinions, attitudes, and behaviours, and can be measured by documenting objective events or relying on subjective perceptions of events [1].

Discrimination can have a significant impact on the lives of those affected. The stress of perceived discrimination negatively affects mental and physical health, and may increase the likelihood of risky health behaviors [2–6]. Perceived discrimination is shown to have a negative impact on help seeking, access to care, poverty, and social marginalization [7]. Within healthcare settings, discrimination by healthcare providers can function as a key barrier to obtaining needed services, resulting in avoidance or delays in treatment seeking, underdiagnosis and mistreatment, nonadherence with or discontinuation of treatment, and poor treatment outcomes [8–11]. A recent Canadian review of discrimination in healthcare noted that negative health outcomes were evident “even under conditions of equal access to medical care” [12] p.19.

Recent meta-reviews of discrimination research from the U.S. highlight that the preponderance of studies to date focus on race- and gender-based discrimination [13, 14]. Yet for certain marginalized populations, discrimination from other defining group characteristics – such as homelessness, poverty, mental illness, and substance use – may have an equal if not greater impact than discrimination due to race or gender [15–18]. In fact, despite the weighty, negative impacts of discrimination in healthcare settings, surprisingly little is known about the different kinds of discrimination that marginalized individuals encounter, besides those due to race. Individuals who belong to several disadvantaged groups may suffer aggravated and specific forms of discrimination in consequence. Furthermore, researchers have noted the potential additive effects of stigma and discrimination, though this is less frequently explored [19].

Homeless persons with mental illness are among the most marginalized patient groups. Adults experiencing homelessness tend to report high levels of unmet health needs [20, 21], due in part to numerous access barriers, including perceived discrimination in healthcare settings [22]. Stigma and discrimination due to mental illness can have more devastating and long-lasting effects on the individual than the mental disorder itself [23] and perpetuate a cycle of impoverishment and disability [24, 25]. While the link between homelessness and mental illness is well documented [25], the experiences of discrimination among homeless people with mental illness within healthcare settings are not well known or understood [10].

This study aims to address some of these gaps in the literature by exploring experiences of discrimination in healthcare settings in an ethnically diverse sample of homeless adults with mental illness in a large urban centre. We used the Aday Framework for Studying Vulnerable Populations to guide this work [26]. According to this framework, vulnerable populations are defined as those groups of individuals who have a greater-than-average risk of poor health by virtue of their marginalized social characteristic(s) such as race/ethnicity, gender, age, socioeconomic status, mental health, or(and) disability [26]. Perceived discrimination was assessed across three domains: (i) homelessness and poverty; (ii) mental health, alcohol and drug-related issues; and (iii) race or ethnicity or skin color. We explored the prevalence of perceived discrimination in the previous 12 months and its association with socio-demographic characteristics, self-reported measures of psychiatric symptomatology and substance use, and Emergency Department (ED) utilization. Finally, we examined whether perceiving certain types of discrimination was associated with increased likelihood of perceiving other kinds of discrimination in this sample of homeless adults with mental illness who experience multiple disadvantages.

## Methods

### Study sample

This study used data from the Toronto site of the At Home/Chez Soi project, a randomized controlled trial of Housing First for homeless persons with mental illness in five cities across Canada. Details of the Toronto site recruitment and study design have been described previously [27]. Eligibility criteria for the randomized controlled trial included: (1) legal adult status (≥18 years old); (2) absolute homelessness or precarious housing (for definitions see [28]); and (3) presence of a mental illness with or without a co-existing substance use disorder, as determined by the MINI International Neuropsychiatric Interview at study entry [29]. Respondents were excluded from the study if they were receiving intensive case management or assertive community treatment at the time of recruitment, had no legal immigrant or refugee status in Canada, or were relatively homeless (individuals who inhabit spaces that do not meet basic health and safety standards, such as living in overcrowded or hazardous conditions, or couch surfing)^a^. In addition, this study excluded individuals for whom interviewers gave poor subjective confidence scores that indicated unreliable responses. Our final study sample included 550 individuals.

All participants gave written informed consent for the study. Structured interviews were conducted by trained research assistants. The study has been registered with the International Standard Randomized Control Trial Number Register (ISRCTN 42520374) and was approved by the Research Ethics Board at St. Michael’s Hospital in Toronto, Canada.

### Measures

#### Dependent variables

Outcome variables measured perceived discrimination experiences in healthcare, and were adapted from the Toronto Street Health survey, developed for homeless participants [30, 31]. Questions about perceived discrimination covered a wide range of discriminatory perceptions. Respondents were asked: “Thinking specifically of all experiences you have had with healthcare visits in the last 12 months, have you ever felt that the doctor or healthcare staff you saw judged you unfairly or treated you with disrespect because of …” and reported on various perceived experiences of discrimination, such as homelessness, poverty, mental health or substance use problems, ethnicity, race and skin color. The responses for each variable were coded as “yes” or “no”. Experiences of perceived discrimination were subsequently grouped into three categories based on conceptual relatedness and significant correlations between the variables within each domain. The three discrimination domains included: discrimination due to (1) homelessness or poverty (yes = 1; no = 0), (2) mental health, alcohol and drug-related issues (yes = 1; no = 0), and (3) race or ethnicity or skin color (yes = 1; no = 0).

#### Independent variables

In light of conceptual understandings and prior research, our study controls for age, sex, race/ethnicity, income, and duration of homelessness. The self-report socio-demographic questionnaire in this study was based on the Demographics, Service Use & Housing History questionnaire adapted from the 2006 Census of Canada [32], the Toronto Board of Education Student Census [33], and the Community Mental Health Evaluation Initiative [34]. Housing categories included living on the street, temporary/unstable residences (<6 months and unprotected tenancy rights), stable residences (>6 months/no current plans to move and/or tenancy rights), emergency shelters/crisis housing, and institutions (e.g., detox facility, nursing home/long-term care facility, addictions treatment or recovery residential program, hospital, or jail). Homelessness was defined as currently having no fixed place to stay for more than seven nights and little likelihood of obtaining accommodation in the upcoming month or being discharged from an institution, prison, jail or hospital with no fixed address [28]. Duration of homelessness was dichotomized using a median split (<3 vs. ≥3 years). The race/ethnicity variable included three categories: White, Black, and Other racial/ethnic minority, consisting of individuals with Asian, Middle Eastern, Aboriginal, Latin American, and mixed racial/ethnic background.

Service use was assessed by self-report. Having a regular medical doctor was assessed by asking: “Do you have a regular medical doctor? By regular medical doctor we mean a family doctor or GP who is familiar with you and your medical history”. A variable on usual source of outpatient care was assessed by asking: “Is there a place that you usually go to when you are sick or need advice about your health?” Participants selected from a list of various inpatient and outpatient services. Presence of provincial health insurance was assessed by the question: “Do you have a provincial health card number?” Unmet needs were evaluated by asking: “In the past 6 months, was there ever a time when you felt that you needed healthcare but you didn’t receive it?” These variables have been previously used by our team to assess barriers to accessing care among vulnerable populations.

Mental health symptoms were assessed by the Modified Colorado Symptom Index (CSI) [35]. The CSI includes 14-items that assess the presence and frequency of psychiatric symptoms within the past month on a 5-point ordinal scale [35–38]. A higher score indicates a higher level of psychiatric symptomatology. Internal consistency (α =0.90) of the modified CSI in this sample was similar to the value reported by Conrad et al. [37]. Missing values on the CSI (8%) were replaced with average item scores for respondents who answered at least 9 of the 14 items. An overall score of 30 was set as a cutoff for clinically significant symptoms denoting the need for further psychiatric assessment [36–38].

Delusions and erroneous beliefs that are present in psychotic conditions may interfere with the individual’s ability to think clearly, distinguish symptoms from reality, and relate to others, which may result in misinterpretation of experiences related to discrimination. Thus, presence of psychosis was used as a covariate (control variable) to account for the possible effect of psychosis on the perceived experiences of discrimination. Based on the interviewers’ impressions and comments, the study excluded individuals who were believed to provide unreliable responses (e.g., due to a lack of cooperation or intoxication), effect of other sources of variation. Presence of a psychotic disorder was determined by DSM-IV criteria on the Mini International Neuropsychiatric Interview (MINI) 6.0 [39, 40]. The MINI has demonstrated excellent inter-rater and test-retest reliability, and good validity against the Composite International Diagnostic Interview [41] and Structured Clinical Interview for DSM-IV-TR Axis I [42].

Severity of substance use problems was evaluated using the 5-item Substance Disorder Screener (SDScr) of the Global Assessment of Individual Need Short Screener (GAIN-SS). GAIN-SS offers a validated and standardized clinical and research assessment of alcohol and substance use disorders based on the DSM-IV [43, 44]. The GAIN SDScr is composed of 5 items. Counts were generated for the number of lifetime substance use symptoms, with higher scores representing greater severity of substance use problems over the life course (in the past month, 2–12 months ago and 1 and more years ago). The counts were stratified into low (0 symptoms), moderate (1–2 symptoms), and high (3 or more symptoms) severity [43]. The reliability of GAINS SDScr in this sample was 0.90.

The number of hospital emergency department (ED) visits in the past 6 months was used as a proxy for frequency of contacts with the healthcare system. Homeless people have substantially higher rates of ED use compared to the general population [45] and hospital EDs serve as the primary means of entry into the healthcare system for homeless people, who have limited access to primary care providers in many service contexts [46, 47]. Therefore, ED utilization can provide useful proxy information about contacts and experience with the healthcare system. Number of ED visits was assessed by two questions: “In the past 6 months, have you been to a hospital emergency room?” and “Approximately how many emergency room visits did you have in total?”.

### Analysis

First, we conducted descriptive analyses to determine the overall prevalence of discrimination. Data analysis involved both univariate and multivariable logistic regression techniques. Variables that could be associated with perceived discrimination were selected *a priori*, guided by previous research and Aday’s framework for studying vulnerable populations [26]. The following variables were considered as possible predictors of perceived discrimination: age (years/10), sex (male or female), race (White, Black, or other), monthly income (<$500, $500-$999, or ≥ $1,000), duration of homelessness (<3 or ≥3 years), clinically significant psychiatric symptoms (CSI score: <30 or ≥30), presence of psychotic disorder (MINI: yes or no), severity of lifetime substance abuse (GAIN SDScr: no/low, moderate, or high), hospital ED visits in the past 6 months (no visits, 1 visit, or ≥ 2 visits). For pairs of highly correlated independent variables, only one was chosen; immigrant status (foreign born vs. Canadian born) was not included because it is significantly associated with race/ethnicity. Eight transgender/transsexual individuals were grouped with the “male” category to allow for cell counts above five in the bivariate analysis and to reflect the predominant male biological gender of these individuals. For the multivariable regression models, three domains of discrimination were used as dependent variables to evaluate the effect of a select group of predictor variables on the probability of expressing perceived discrimination. Variables that were associated with outcomes with a *P* value of ≤ .20 in the univariate analysis were simultaneously entered into multivariable logistic models. Presence of psychotic disorder was entered in all multivariable models as a control variable. Interaction terms were not examined because the main effects model fitted adequately and we did not have a theory-based proposition for expecting significant interactions. Multicollinearity between independent variables was assessed and was determined not to be an issue in any of the models. The fit of the models was assessed using Hosmer and Lemeshow’s goodness of fit statistic.

None of the variables contained 5% or more missing values, except for the CSI variable, which contained 8% of missing data. Missing data for the CSI were replaced with individual participants’ average item score as long as the respondent answered at least 9 of the 14 items. A complete case analysis was used for handling the rest of the missing data in multivariable models. Reliability statistics of the scales were assessed using the Cronbach alpha. All analyses were done using IBM SPSS Statistics v.20 for Windows. The significance level of *P* < 0.05 was used throughout the study, though borderline level of significance of *P* <0.06 was also reported.

## Results

The sample’s socio-demographic characteristics are presented in Table 1. Roughly 80% of the respondents had clinically significant mental health symptoms (CSI score ≥30). Nearly two-thirds of the sample had experienced high severity of lifetime substance use problems. Similar to previous studies that reported high rates of concurrent psychiatric and substance use disorders among homeless people [48, 49], as many as 54% (not reported in Table 1) of our sample had both severe psychiatric illness and severe substance use problems. Of the total sample, 36% of individuals were found to have a current psychotic disorder. Although the majority of respondents had a regular medical doctor (66%), a usual source of outpatient care (90%), and a provincial health card (93%), 40% reported unmet healthcare needs.

**Table 1:** **Characteristics of homeless adults with mental illness at the Toronto site of the At Home/Chez Soi project (** ***N*** **= 550)**

Characteristic		Total N (%)
Age, y	<30 years old	131 (23.8)
	30-39 years old	129 (23.5)
	40-49 years old	176 (32.0)
	≥50 years old	114 (20.7)
Male^£^		384 (69.8)
Race^§^	White	194 (35.3)
	Black	190 (34.5)
	Other ethnic minority	166 (30.2)
Monthly income, CAD	<$500	198 (36.0)
	$500-$999	233 (42.4)
	≥$1,000	118 (21.5)
Homeless for ≥3 years		287 (52.2)
Immigrant		251 (45.6)
Has a regular medical doctor		360 (65.5)
Has a usual source of outpatient care		492 (89.5)
Has a provincial health card		512 (93.1)
Unmet healthcare needs		218 (39.6)
CSI ≥30^†^		427 (77.6)
Psychotic disorder^‡^		200 (36.4)
GAIN SDScr (lifetime)	No or low severity	96 (17.5)
	Moderate severity	102 (18.5)
	High severity	342 (62.2)
Hospital ER visits in the past 6 months	No visits	223 (40.5)
	1 visit	141 (25.6)
	≥ 2 visits	178 (32.4)

Column totals may not add to the total sample size because of missing data.

^£^Male category includes transgender/transsexual individuals.

^§^Other ethnic minority category included individuals with Asian, Middle Eastern, Aboriginal, Latin American, and mixed racial/ethnic background.

^†^CSI ≥30, score ≥30 on the Colorado Symptom Index indicates psychiatric disability and presence of clinically significant mental symptoms.

^‡^MINI v. 6.0; the Mini International Neuropsychiatric Interview 6.0.

GAIN SDScr; Global Assessment of Individual Need Short Screener. Counts are triaged into low (0 symptoms), moderate (1-2 symptoms), and high (3 or more symptoms) severity symptoms.

The prevalence of the various types of discrimination experienced within healthcare settings in the past 12 months is presented in Table 2. Overall, 42% of respondents reported at least one form of perceived discrimination (not reported in Table 2). After grouping variables in the three categories of interest, the most prevalent forms of perceived discrimination were due to mental illness/substance use (33%) and homelessness/poverty (30%). Only 20% of non-White and 15% of the total participants reported discrimination due to race/ethnicity/skin color. Interestingly, those who reported discrimination due to homelessness/poverty were 32 times more likely to report discrimination due to mental illness/substance use (odds ratio (OR) =32.0, 95% Confidence Interval (CI) =19.3-53.2, P < .001), and 10 times more likely to report discrimination due to race/ethnicity/skin color (OR = 10.3, 95% CI 6.0-17.6, P < .001). Those who have experienced discrimination due to mental illness/substance use were almost 9 times more likely to report discrimination due to race/ethnicity/skin color (OR = 8.8, 95% CI 5.1-15.3, P < .001).

**Table 2:** **Prevalence of perceived experience of discrimination within healthcare settings among homeless adults with mental illness at the Toronto site of the At Home/Chez Soi project (** ***N*** **= 550)**

Discrimination due to:	Percentage distribution, N (%)
Homelessness or poverty	167 (30.4)
Homelessness	147 (26.7)
Poverty	134 (24.4)
Mental health, alcohol and drug-related issues	179 (32.5)
Mental health issues	131 (23.8)
Use of alcohol or drugs	112 (20.4)
Race or ethnicity or skin color	84 (15.3)
Race	65 (11.8)
Ethnicity	68 (12.4)
Skin color	59 (10.7)

### Discrimination due to poverty or homelessness within healthcare settings

Table 3 shows the results of multivariable analysis models predicting the unadjusted and adjusted odds ratios with 95% confidence intervals of individuals perceiving discrimination in the three domains. In the unadjusted analysis, White race (as compared to Black race), higher income, ≥3 years duration of homelessness, presence of clinically significant mental health symptoms, moderate to severe substance use, and any ED visit in the past 6 months were significant determinants of perceived discrimination due to poverty or homelessness. In the adjusted model, significant independent predictors of discrimination due to poverty or homelessness were duration of homelessness ≥3 years, clinically significant mental health symptoms (borderline level of significance at p < .06), moderate to severe substance use problems and ≥2 ED visits in the past 6 months.

**Table 3:** **Associations between participants’ characteristics and type of perceived discrimination within healthcare settings**

Explanatory variables		Discrimination due to poverty or homelessness		Discrimination due to mental illness or alcohol or drugs		Discrimination due to race or ethnicity or skin color	
		Unadjusted OR	Adjusted OR ^1^	Unadjusted OR	Adjusted OR ^2^	Unadjusted OR	Adjusted OR ^3^
Age^§^, years, mean (SD)		.95 [.81-1.12]	**-**	**.78 [.67-.92]** ^**^	**.70 [.58-.85]** ^***^	.88 [.71-1.08]	**-**
Male^£^		1.10 [.74-1.64]	**-**	.83 [.56-1.21]	**-**	1.13 [.68-1.90]	**-**
Race	White (ref)	1.0	1.0	1.0	1.0	1.0	1.0
	Black	**.53 [.34-.83]** ^**^	.72 [.44-1.20]	**.39 [.25-.60]** ^***^	**.46 [.28-.77]** ^**^	**3.13 [1.66-5.91]** ^***^	**3.96 [1.99-7.88]** ^***^
	Other ethnic minority	.79 [.51- 1.23]	.94 [.58-1.55]	**.54 [.35-.85]** ^**^	**.53 [.32-.89]** ^*^	**2.65 [1.37-5.12]** ^**^	**3.22 [1.61-6.42]** ^**^
Monthly income, CAD	<$500 (ref)	1.0	1.0	1.0	1.0	1.0	**-**
	$500-$999	**1.52 [.99-2.33]** ^**¶**^	1.25 [.78-1.99]	**1.78 [1.17-2.72]** ^**^	1.47 [.91-2.37]	1.30 [.77-2.21]	**-**
	≥$1,000	**1.78 [1.08-2.92]** ^*^	1.42 [.82-2.44]	**2.00 [1.22-3.28]** ^**^	1.50 [.86-2.62]	1.03 [.56-2.09]	**-**
Homeless for ≥3 years		**1.80 [1.23-2.61]** ^**^	**1.64 [1.08-2.48]** ^*^	**1.55 [1.07-2.24]** ^*^	**1.60 [1.04-2.47]** ^*^	1.34 [.83-2.16]	**-**
CSI^†^ ≥30		**2.35 [1.41-3.93]** ^**^	**1.75 [.99-3.10]** ^**¶**^	**2.92 [1.73-4.92]** ^***^	**1.97 [1.11-3.52]** ^*^	**1.94 [.99-3.79]** ^**¶**^	**2.16 [1.03-4.55]** ^*^
GAIN SDScr^║^	No/low (ref)	1.0	1.0	1.0	1.0	1.0	1.0
	Moderate	**2.45 [1.18-5.09]** ^*^	**2.54 [1.17-5.50]** ^*^	1.65 [.82-3.33]	1.27 [.59-2.72]	**3.39 [1.44-7.99]** ^**^	3.76 [1.56-9.09]**
	High	**3.58 [1.91-6.69]** ^***^	**2.41 [1.21-4.81]** ^*^	**3.25 [1.82-5.81]** ^***^	1.49 [.77-2.87]	1.92 [.88-4.21]	1.85 [0.81-4.21]
Psychotic disorder^‡^		.82 [.56-1.20]	1.04 [.68-1.60]	**.58 [.39-.86]** ^**^	.76 [.48-1.18]	1.11 [.69-1.80]	1.02 [0.60-1.73]
ED visits in the past 6 months	No visits (ref)	1.0	1.0	1.0	1.0	1.0	1.0
	1 visit	**1.64 [1.01-2.66]** ^*^	1.55 [.92-2.61]	**2.29 [1.41-3.70]** ^**^	**1.81 [1.07-3.07]** ^*^	**2.11 [1.16-3.86]** ^*^	**2.68 [1.41-5.09]** ^**^
	≥ 2 visits	**2.62 [1.69-4.08]** ^***^	**2.07 [1.28-3.35]** ^**^	**3.43 [2.20-5.35]** ^***^	**2.57 [1.57-4.20]** ^***^	**2.02 [1.13-3.58]** ^*^	**2.55 [1.37-4.76]** ^**^

CI, confidence interval; OR, Odds Ratio (95% CI). Presence of psychotic disorder was entered in all multivariable models as a control variable.

^§^Age is measured in decades in logistic regression analyses.

^£^Male category includes transgender/transsexual individuals.

^†^CSI ≥30, score ≥30 on the Colorado Symptom Index indicates psychiatric disability and presence of clinically significant mental symptoms.

^║^GAIN SDScr, Substance Disorder Screener. Life time severity counts are triaged into low (0 symptoms), moderate (1-2 symptoms), and high (3 or more symptoms) severity symptoms.

^‡^MINI v. 6.0; the Mini International Neuropsychiatric Interview 6.0.

1 - Model coefficients: χ2 (11, n=509) = 47.79, P < .001; Hosmer&Lemeshow test: χ2 (8, n=509) = 10.9, *p* =.21.

2 - Model coefficients: χ2 (12, n=504) = 80.35, P < .001; Hosmer&Lemeshow test: χ2 (8, n=504) = 4.91, *p* =.77.

3 - Model coefficients: χ2 (8, n=519) = 41.27, P < .001; Hosmer&Lemeshow test: χ2 (8, n=519) = 9.65, *p* =.29.

*p<.05; **p<.01; ***p<.001; Borderline significance at *p*<.06.

### Discrimination due to mental illness or substance use within healthcare settings

Participant characteristics significantly associated with reporting discrimination due to mental illness or substance use were younger age, being White (as opposed to Black or other ethnic minority), having higher income, being homeless for ≥3 years, having clinically significant mental health symptoms, high severity of substance use problems, absence of psychotic disorder, and reporting any ED visits in the past 6 months (Table 3). In the adjusted model, significant predictors of perceived discrimination due to mental illness/substance use were younger age, White race, being homeless for ≥3 years, having clinically significant mental health symptoms, and reporting any ED visits in the past 6 months.

### Discrimination due to race or ethnicity or skin color within healthcare settings

The unadjusted analysis showed that being non-White, having clinically significant mental health symptoms, moderate lifetime severity of substance use (as compared to no or low severity of substance use problems) and having any ED visits in the past 6 months significantly increased the odds of experiencing discrimination due to race/ethnicity/skin color in the past 12 months (Table 3). All the associations remained significant in the adjusted model. We used sensitivity analysis to examine robustness of the predictors for the race/ethnicity/skin color discrimination in the subsample of non- White participants (n = 329) as their experiences of this form of discrimination will be inherently different from the White counterparts (see Table 4). In the sensitivity analysis, no significant differences were observed between Blacks and other ethnic minorities with regard to the discrimination due to race/ethnicity/skin color within healthcare settings. Both moderate lifetime severity of substance use and having any ED visits in the past 6 months significantly increased the likelihood of experiencing discrimination due to race/ethnicity/skin color in the past 12 months among non-Whites. The association of clinically significant mental health symptoms with the race/ethnicity-based discrimination was, however, not significant in this sub-sample.

**Table 4:** **Associations between participants’ characteristics and race/ethnicity based discrimination within healthcare settings among non-White participants**

Explanatory variables		Discrimination due to race or ethnicity or skin color (n = 329)	
		Unadjusted OR	Adjusted OR ^3^
Age^§^, years, mean (SD)		.94 [.75-1.18]	**-**
Male^£^		1.07 [.61-1.90]	**-**
Race	Black (ref)	1.0	-
	Other ethnic minority	.84 [.50-1.44]	**-**
Monthly income, CAD	<$500 (ref)	1.0	1.0
	$500-$999	1.80 [.98-3.32]	1.54 [.79-3.00]
	≥$1,000	1.96 [.94-4.09]	1.87 [.83-4.19]
Homeless for ≥3 years		1.57 [.92-2.68]	1.54 [.85-2.80]
CSI^†^ ≥30		1.69 [.84-3.41]	1.67 [.75-3.70]
GAIN SDScr^║^	No/low (ref)	1.0	1.0
	Moderate	**3.61 [1.43-9.13]** ^**^	3.66 [1.39-9.61]**
	High	**2.82 [1.20-6.60]** ^*^	1.75 [.70-4.38]
Psychotic disorder^‡^		.97 [.57-1.67]	1.05 [0.58-1.89]
ED visits in the past 6 months	No visits (ref)	1.0	1.0
	1 visit	**2.68 [1.38-5.19]** ^*^	**3.17 [1.56-6.48]** ^**^
	≥ 2 visits	**2.58 [1.34-4.96]** ^*^	**2.27 [1.13-4.55]** ^*^

The adjusted model includes variables that were associated with the outcome variable at the level of P ≤.20 in the univariate analysis and presence of psychotic disorder as a control variable.

CI, confidence interval; OR, Odds Ratio (95% CI).

^§^Age is measured in decades in logistic regression analyses.

^£^Male category includes transgender/transsexual individuals.

^†^CSI ≥30, score ≥30 on the Colorado Symptom Index indicates psychiatric disability and presence of clinically significant mental symptoms.

^║^GAIN SDScr; Substance Disorder Screener. Counts are triaged into low (0 symptoms), moderate (1-2 symptoms), and high (3 or more symptoms) severity symptoms.

^‡^MINI v. 6.0; the Mini International Neuropsychiatric Interview 6.0.

Model coefficients: χ2 (9, n=329) = 27.24, P < .01; Hosmer&Lemeshow test: χ2 (8, n=329) = 4.28, *p* =.83.

*p<.05; **p<.01; ***p<.001.

## Discussion

Our findings suggest that among ethnically diverse homeless adults with mental illness in a large urban center in Canada, perceived discrimination in healthcare settings is common. Perceptions of discrimination due to homelessness/poverty (30.4%) and mental health/substance use problems (32.5%) were more frequently reported than discrimination due to race/ethnicity/skin color (15.3%).

Our results on the prevalence of perceived discrimination based on mental illness and alcohol or substance use are not surprising. Previous research has shown that among adults with serious mental illness, the most common grounds for self-reported discrimination is mental disability rather than discrimination due to other characteristics, such as race, gender, sexual orientation, or physical disability [15, 18]. In 2004, the Standing Senate Committee on Social Affairs, Science and Technology of Canada recognized the widespread stigma and discrimination associated with mental illness in all walks of Canadian society and acknowledged that combating discrimination “requires a multi-pronged effort sustained over a long period of time” [50] (Chapter 4, Section 4.1, p. 25).

We similarly found a high prevalence of perceived discrimination due to homelessness and poverty in healthcare settings in our sample. Discrimination towards homeless people, particularly those with concurrent mental illness, has not been widely studied. Homelessness as a marker of social status and extreme poverty may lead to more perceived discrimination than race/ethnicity. Not surprisingly, we found a higher prevalence of discrimination due to poverty or homelessness among individuals who have been homeless for three years or longer, suggesting that stigmatizing attributes of homelessness become more salient over time.

Although over two thirds of sample was non-White, discrimination due to race/ethnicity/skin color was reported by only 15% of the participants. Previous research has shown that people living with serious mental illness tend to report less discrimination due to race compared to discrimination associated with mental disability [15, 18]. Studies have also documented that perceived everyday racial discrimination is significantly underreported by individuals who belong to lower socioeconomic status [51, 52]. Another plausible explanation of these findings is that most race-based discriminatory encounters in Canada today are subtle, elusive, or systemic relative to traditionally overt forms, and may be more difficult for individuals to identify or reconcile [53, 54].

Individuals who experienced discrimination due to homelessness/poverty were significantly more likely to report discrimination due to mental illness/substance use. Stigma and discrimination due to mental illness are known to perpetuate a cycle of impoverishment [24, 25]. Mental illness and homelessness may be two highly salient and overlapping social identities in our sample. A higher reporting of discrimination due to poverty/homelessness and mental illness/substance use among individuals with mental illness who have been homeless for three years or longer confirms the interrelatedness of these marginalized social statuses. Furthermore, a considerable psychosocial stress associated with homelessness makes it difficult to disentangle issues experienced by homeless people with mental illness from those of the homeless population at large [55]. A greater understanding of perceived discrimination related to the unique experiences of homeless people will require research that looks at multiple types – and compound effects – of discrimination and its health effects.

The self-reported experiences of discrimination across all three domains were significantly associated with the severity of mental health problems and ED utilization in adjusted models. Previous research has suggested that the stigma of mental illness may be perceived differently depending on the severity of mental illness [56]. Severity of mental illness is accompanied by different characteristics of psychosocial disability noticeable to the public (e.g. violent behaviour or homelessness). Therefore, the lay perception of people with mental illness is influenced by their psychosocial disabilities, which have differential impact on the degree of perceived stigma accompanying the disease [56]. A lack of respectful treatment by general practitioners and ED clinicians have been reported as the most common complaint among people with mental illness in Canada [57].

Individuals with more severe lifetime substance use problems were significantly more likely to report discrimination due to homelessness/poverty and race/ethnicity/skin color in healthcare settings, even after adjusting for socio-demographic characteristics, severity of mental illness and frequency of ED visits.

Interestingly, the severity of lifetime substance use problems was not associated with the likelihood of perceived discrimination due to mental illness/substance use. It has been suggested that substance use is a way of coping with stressful life events and the chronic stressors of homeless life [58] and racism [59]. Substance use itself in this population group may not be perceived as stigmatizing, but rather a component of the homeless subculture [60]. In this study, discrimination due to mental illness/substance use was more prevalent among younger Whites. These findings point to a greater need for additional consideration of race and age in future research on perceived stigma and discrimination.

Individuals who perceived discrimination due to race/ethnicity/skin color within healthcare settings were more likely to be non-White, have moderate severity of lifetime substance use problems, and report ED use in the past 6 months. Duration of homelessness, as expected, did not affect the likelihood of reporting race/ethnicity-based discrimination. In our study, sensitivity analyses showed that perceived discrimination due to race/ethnicity/skin color may be differentially biased among White participants compared to non-Whites, and likely explained the original association between the race/ethnicity-based discrimination and CSI score in the total sample.

Overall, our study revealed that individuals who experienced discrimination in one domain were more likely to report discrimination in the other two domains and supports the need for an intersectional theoretical framework [55, 61–64] in future research. A cumulative effect of various types of discrimination could be especially detrimental for individuals who belong to multiple stigmatizing social identities, like those in our study sample. Future research is needed to examine the cumulative impact of multiple dimensions of perceived discrimination on individual health and wellbeing.

High unmet healthcare needs despite relatively good indicators of access to healthcare (having a usual source of outpatient care, a regular medical doctor and a provincial health card) identified in this population suggest that discrimination may be an important determinant of access to healthcare among disadvantaged patients. High prevalence of unmet healthcare needs in this sample is supported by previous studies of homeless individuals with mental illness [20, 21]. Past research has also established that health service underutilization among homeless people is related to perceived stigma and negative perceptions of service staff (e.g. [9, 10, 65–70]). Intense emotional responses to these experiences can greatly influence homeless individuals’ willingness to seek healthcare in the future [10]. Focused efforts to assess and improve healthcare providers’ attitudes towards homeless people have been proposed to address this issue [71]. Future research should further explore the associations between perceived discrimination and unmet healthcare needs within the setting of universal health coverage and good healthcare access.

Important limitations of our study are the cross-sectional and observational nature of the data that precludes us from making inferences regarding cause and effect. It is possible that our research has the same-source bias. This could have generated erroneous associations due to the correlated measurement error or because the outcome affects the perception (e.g. self-reported ED visits and perceived discrimination in healthcare settings). Furthermore, the recall of healthcare utilization among homeless persons has been reported as less accurate than the general public [72]. In our study perceived discrimination was a subjective measure rather than observable acts of discrimination. We did not collect information on the frequency and extent of perceived discrimination, e.g. minor incidents or serious assaults. In addition, a larger representative sample of sexual minorities will be needed to allow comparisons of transgender/transsexual individuals with other groups*.*

The strengths of the study include the use of a large sample of racially diverse homeless adults with mental illness, and rich information on perceptions of discrimination and measures of mental health and substance use, which allowed us to address some of the gaps and limitations in previous research. We explored perceived discrimination within healthcare settings related to poverty, homelessness, mental illness, substance use, race, ethnicity, and skin color, which are particularly relevant for this vulnerable population. To the best of our knowledge, this is the first such study in Canada. The associations we established, while not necessarily causal, help build a detailed picture of discrimination experienced by homeless people in contemporary Canada. Future research, preferably using longitudinal data, is needed to provide a better assessment of the direction and strength of the causal paths in the reciprocal relationships between perceived discrimination within healthcare settings, aspects of mental health status, and healthcare utilization.

## Conclusion

In our sample of homeless individuals with severe mental illness, perceived discrimination due to marginalized social status was associated with a greater severity of lifetime substance abuse and mental health problems and more frequent ED use. There is an urgent need for public health policies and strategies geared toward minimizing discrimination associated with mental illness and devalued socio-economic status in order to enhance equitable access to medical treatment and other health services.

## Endnotes

^a^A more detailed definition is described in Goering et al. (2011).

## Abbreviations

CSI: Colorado Symptom Index
ED: Emergency Department
GAIN-SS: Global Assessment of Individual Need Short Screener
SDScr: Substance Disorder Screener
SES: Socio-economic status
MINI: Mini International Neuropsychiatric Interview.
